# Molecular Aspects of Rare Coagulation Factor Deficiencies

**DOI:** 10.7759/cureus.89102

**Published:** 2025-07-31

**Authors:** Hajar Tourbih, Asma Harrach, Hanaa Bencharef, Hind Dehbi, Bouchra Oukkache

**Affiliations:** 1 Faculty of Medicine and Pharmacy, Hassan II University, Casablanca, MAR; 2 Hematology Laboratory, Ibn Rochd University Hospital Center, Casablanca, MAR; 3 Medical Genetics Laboratory, Ibn Rochd University Hospital, Casablanca, MAR

**Keywords:** coagulation, diagnosis, hemorrhage, mutation, rare deficiency

## Abstract

Rare coagulation factor deficiencies are inherited disorders affecting factors I, II, V, VII, X, XI, XIII, and combined forms. They mainly present with bleeding symptoms of variable severity. These deficiencies show significant clinical heterogeneity, with no consistent correlation between factor levels and bleeding intensity. Prevalence is higher in populations with high consanguinity rates. Understanding relies on the study of genetic and molecular mechanisms.

Diagnosis is based on hemostasis testing, including prothrombin time (PT), activated partial thromboplastin time (aPTT), and factor activity assays. In case of abnormalities, Molecular analysis is undertaken using genomic DNA extracted from peripheral blood samples. Depending on the clinical context and the level of detail required, sequencing is performed using either Sanger sequencing or next-generation sequencing (NGS) platforms, enabling the identification of pathogenic variants responsible for the coagulation disorder. Variant interpretation follows American College of Medical Genetics and Genomics (ACMG)/Association for Molecular Pathology (AMP) guidelines. Large deletions or duplications are assessed via Multiplex Ligation-dependent Probe Amplification (MLPA) or Copy number variations (CNV) analysis.

Molecular analysis revealed a wide range of mutations depending on the deficient factor. Missense mutations are the most common, followed by nonsense, splicing, and insertion/deletion mutations. Some mutations prevent factor production, while others alter its structure or secretion. Each mutation type has a distinct impact on the bleeding phenotype. Genetic characterization improves diagnostic accuracy and guides clinical management. Modern molecular tools have enhanced the understanding of these disorders’ pathophysiology. A multidisciplinary approach is essential for optimal patient care. Ongoing research is key to developing targeted therapies.

## Introduction and background

Coagulation factor deficiencies are a group of inherited bleeding disorders characterized by a reduced activity of one or more proteins essential to the blood clotting cascade. Normally, the coagulation cascade is a highly regulated process that leads to thrombin generation and formation of a stable fibrin clot, which effectively seals vascular injuries and prevents excessive bleeding. In coagulation factor deficiencies, this process is disrupted, resulting in impaired clot formation and an increased risk of hemorrhage [1]. Rare coagulation factor deficiencies (RCFDs) encompass isolated constitutional deficiencies of factors II, V, VII, X, XI, XIII, fibrinogen, vitamin K-dependent factors, and combined FV and FVIII deficiencies [1,2]. Most of these disorders follow an autosomal recessive inheritance pattern, except for some cases like factor XI deficiency and fibrinogen abnormalities, which may have different genetic modes of transmission. Their occurrence is observed in the majority of populations worldwide, manifesting either as homozygous or as a compound double heterozygote. The incidence of these conditions is varied, ranging from approximately 1 in 500,000 for factor VII deficiency, 1 in 1,000,000 for factor I, V, X, and XI deficiency to approximately 1 in 2 to 3 million for factor II and factor XIII deficiencies [3]. The most common deficiencies include factor VII deficiency and factor XI deficiency, which account for approximately two-thirds of pathological cases, while factor XIII deficiencies are less common. The prevalence of these deficiencies shows substantial variation, with a notably higher incidence in specific regions, particularly among populations with a high rate of consanguinity [2].

In Morocco, data on the prevalence and molecular characteristics of these rare deficiencies remain scarce, with few documented cases in the literature. Rare coagulation factor deficiencies can be identified in various clinical settings, including routine hemostatic workups, familial screening, or following bleeding episodes. The clinical presentation is markedly heterogeneous, ranging from asymptomatic individuals to those exhibiting mild bleeding, or, in more severe cases, life-threatening hemorrhages either spontaneous or triggered by trauma or surgery [4]. Notably, there is often a poor correlation between plasma factor levels and the severity of clinical manifestations. The main objective of this review is to summarize current knowledge on the genetic and molecular aspects of rare coagulation disorders.

## Review

Genetic diagnosis to identify mutations in rare clotting factor deficiencies

*Polymerase Chain Reaction* (*PCR)*

The polymerase chain reaction (PCR) is a laboratory technique used to specifically amplify a DNA sequence from minute quantities. The process relies on the use of the enzyme DNA polymerase, which generates multiple copies of a targeted DNA segment. PCR involves several steps: first, the double-stranded DNA is heated to separate it into two single strands (denaturation). Then, the temperature is lowered to allow the primers, small fragments of DNA complementary to the targeted sequences, to bind to the single strands (annealing). Then, the temperature is increased to allow the DNA polymerase to extend the primers and synthesize new DNA strands (elongation). These steps are repeated several times, doubling the amount of DNA with each cycle. This technique is particularly useful when DNA is limited, as it allows the sequence of interest to be amplified sufficiently for further analysis. PCR is widely used in fields such as infectious disease diagnosis, genetic mutation analysis, genetic identification, and genomic research, providing a rapid and accurate method for studying genomes and detecting abnormalities [5].

Benefits: PCR is an extremely powerful and sensitive method used in diverse fields such as infectious disease diagnosis, genetics, biomedical research, and paternity testing. It allows rapid and accurate detection of target DNA, even from very small quantities [6].

Challenges and limitations: PCR presents several challenges that can affect the reliability of its results. First, sensitivity to inhibitors is a major issue: some biological samples may contain substances that interfere with the efficiency of the reaction, compromising the accuracy of the results [7]. Furthermore, interpreting the results can be complex. A positive PCR result simply indicates the presence of the target nucleic acid, but does not necessarily confirm the presence of an infectious agent, such as a virus. Furthermore, results with high cycle thresholds can make interpretation more difficult, as this may indicate a low amount of amplified DNA [8]. Finally, sample variability represents another constraint: the quality and quantity of extracted DNA can vary depending on the sample type and collection conditions, which can affect the reliability of PCR tests. These factors must be taken into account to optimize the use of PCR in diagnostics [9].

Sanger

Sanger sequencing is a DNA sequencing method based on the incorporation of dideoxynucleotides, which halts DNA chain elongation. The resulting fragments are separated by electrophoresis, allowing the nucleotide sequence to be determined. This technique, recognized for its accuracy and ability to analyze targeted regions of the genome, remains a benchmark for detecting point mutations and validating results obtained using other methods. Despite the rise of next-generation sequencing, it is still used for small-scale analyses [10].

Benefits: Sanger sequencing is distinguished by its high accuracy, with very low error rates, making it the gold standard for detecting single-nucleotide variants as well as small insertions and deletions. This method is also particularly effective for targeted applications, such as sequencing specific small regions of the genome. It is ideal for analyzing known familial genetic variants, validating high-throughput sequencing results, and performing single-gene sequencing assays [11].

Challenges and limitations: Although Sanger sequencing offers high accuracy, it has some limitations, including limited read length. This method can only sequence small DNA fragments, which can pose challenges when analyzing large genomic regions [12]. Furthermore, Sanger sequencing can be more expensive and time-consuming than NGS technologies, which allow for parallel sequencing of millions of fragments, making the approach faster and more cost-effective for large-scale analyses [11]. Finally, Sanger sequencing has limited variant detection. It may be less effective at identifying rare variants or low-frequency mutations due to its sensitivity and detection capabilities [13].

*Next-Generation Sequencing* (*NGS)*

NGS encompasses high-throughput sequencing technologies that enable the simultaneous analysis of multiple DNA fragments, providing a comprehensive view of the genome, exome, or specific gene panels [14].

Benefits: NGS enables rapid and accurate diagnosis by facilitating the identification of disease-causing genetic mutations, accelerating the diagnostic process. It also offers comprehensive analysis by allowing the simultaneous examination of multiple genes or even the entire genome, increasing the chances of detecting genetic abnormalities, particularly in complex or atypical cases. Finally, the information generated by NGS can guide therapeutic decisions, enabling personalized treatments based on the specific mutations identified [14].

Challenges and limitations: Interpreting NGS data represents a major challenge due to the large amount of information generated, requiring bioinformatics expertise to differentiate pathogenic mutations from benign variations. Furthermore, NGS may reveal genetic variants whose clinical impact remains uncertain, complicating genetic counseling and medical decision-making. Furthermore, some regions of the genome may be poorly covered, which can lead to incomplete mutation detection. Finally, the incidental discovery of mutations linked to unsought diseases raises ethical questions about how to disclose this information to patients [14].

Molecular genetic identification of rare coagulation factor deficiencies

The identification of genetic abnormalities responsible for rare coagulation factor deficiencies relies on targeted molecular analyses. In this context, genomic DNA is extracted from peripheral blood, and the coding regions (exons) as well as the exon/intron junctions of several genes involved in hemostasis are sequenced. These genes include those encoding coagulation factors such as FII, FV, FVII, FX, FXI, F13A1, F13B, and FI, the fibrinogen chains alpha, beta and gamma (FGA, FGB, FGG), as well as LMAN1, MCFD2, GGCX, and VKORC1. Sequencing is performed using two complementary approaches: Sanger sequencing (with the ABI PRISM 3130 genetic analyzer) and next-generation sequencing (NGS) using the Illumina MiniSeq platform. Results are analyzed using SeqScape and SeqPilot software. The identified variants are interpreted according to the nomenclature guidelines of the Human Genome Variation Society (HGVS), and classified following the criteria of the American College of Medical Genetics and Genomics (ACMG) and the Association for Molecular Pathology (AMP). The pathogenicity of the variants is evaluated by comparison with reference databases such as the Human Gene Mutation Database (HGMD) and ClinGen. In addition, the detection of large genomic rearrangements (deletions or duplications) is performed using MLPA (Multiplex Ligation-dependent Probe Amplification) following the manufacturer’s recommendations with SALSA kits, or by copy number variation (CNV) analysis. This comprehensive molecular approach enables the identification of genetic alterations potentially responsible for rare coagulation factor deficiencies [15].

The diagnostic value of NGS has been recently illustrated by two major cohort studies. A Dutch nationwide study (2024) applied targeted exome sequencing to patients with rare bleeding disorders, achieving a molecular diagnosis in over 80% of cases, including ~10% novel mutations [16]. Similarly, a large international study (EAHAD 2025), involving 527 patients from 19 countries, identified 261 variants, 12% of which were previously unreported [17]. These findings highlight the global relevance of NGS and support its systematic use in the diagnostic workflow.

Pathophysiological, genetic, and molecular aspects of rare coagulation factor deficiencies

Rare coagulation factor deficiencies are mostly inherited in an autosomal recessive manner. The molecular characterization of these rare coagulation factor deficiencies is based on the identification of mutations in the genes encoding the corresponding factors (Table 1), typically performed through DNA sequencing methods such as next-generation sequencing (NGS). However, some exceptions exist, such as the combined deficiency of FV and FVIII, linked to mutations in Multiple Coagulation Factor Deficiency Protein 2 (MCFD2) and Lectin, Mannose-Binding 1 (LMAN1), or the combined deficiency of vitamin K-dependent procoagulant proteins (FII, FVII, FIX, and FX), caused by mutations in gamma-glutamyl carboxylase (GGCX) and vitamin K epoxide reductase complex subunit 1 (VKORC1). Mutations responsible for rare coagulation factor deficiencies are classified into two categories: those completely suppressing the production or secretion of the factor and those producing an abnormal protein, partially or totally secreted, but with reduced activity. The main databases listing these mutations are the International Registry of Rare Bleeding Disorders (RBDD) and the registry of the International Society on Thrombosis and Haemostasis (ISTH), both of which provide similar information. Missense mutations are the most frequent (50-80%), with the exception of LMAN1 variants, where insertions/deletions predominate (50%). Insertion/deletion mutations also concern 20% to 30% of genetic variations in the fibrinogen, FV, MCFD2, and FXIII genes. Splicing and nonsense mutations represent 5% to 15% of all mutations identified in all coagulation factors, reaching 20% ​​in the LMAN1 gene. Mutations located in the untranslated regions (3’ and 5’ UTR) are rare (<5%) and mainly concern the fibrinogen, FVII, FXI and FXIII loci, although their functional impact is often uncertain [18].

**Table 1:** Mutations causing rare bleeding disorders FGA: Fibrinogen alpha chain gene, FGB: Fibrinogen beta chain gene, FGG: Fibrinogen gamma chain gene, F2: Coagulation factor II gene, F5: Coagulation factor V gene, LMAN1: Lectin, Mannose-Binding 1, MCFD2: Multiple Coagulation Factor Deficiency Protein 2, F7: Coagulation factor VII gene, F10: Coagulation factor X gene, F11: Coagulation factor XI gene, F13A1: Coagulation factor XIII A chain gene, F13B: Coagulation factor XIII B chain gene, GGCX: Gamma-glutamyl carboxylase, VKORC1: Vitamin K epoxide reductase complex subunit 1, UTR: Untranslated region.

Deficiency	Gene and chromosome	Missense	Nonsense	Splicing	5’-3’UTR	Deletion/Insertion	Total
Factor I	FGA (4q28)	39 FGA (15.4%)	18 FGA (7.2%)	7 FGA (2.8%)	3 FGA (1.2%)	38 FGA (15%)	105
	FGB (4q28)	39 FGB (15.4%)	7 FGB (2.8%)	5 FGB (1.9%)	3 FGB (1.2%)	8 FGB (3.1%)	62
	FGG (4q28)	67 FGG (26.4%)	4 FGG (1.5%)	7 FGG (2.8%)	1 FGG (0.4%)	7 FGG (2.8%)	86
Factor II	F2 (11q11-q12)	42 (77.8%)	3 (5.5%)	2 (3.7%)	0	7 (13%)	54
Factor V	F5 (1q24.2)	64 (48.5%)	17 (12.9%)	15 (11.3%)	0	36 (27.3%)	132
Factor V + Factor VIII	LMAN1	3 (8.8%)	7(20.6%)	7(20.6%)	0	17 (50%)	34
	MCFD2	11(50%)	1 (4.5%)	4(18.2%)	0	6 (27.3%)	22
Factor VII	F7 (13q34)	151(62.2%)	19(7.8%)	28(11.5%)	15(6.2%)	30 (12.3%)	243
Factor X	F10 (13q34)	84 (80%)	2 (1.9%)	8 (7.6%)	0	11 (10.5%)	105
Factor XI	F11 (4q35.2)	154 (70%)	23(10.5%)	18 (8.2%)	2 (1%)	23 (10.5%)	220
Factor XIII	F13A1 (6p24-p25)	57 (47.1%)	11 (9.1%)	17 (14%)	1 (1%)	35 (29%)	121
	F13B (1q31-q32.1)						
Vitamin K–dependent factors	GGCX	6 (60%)	0	3 (30%)	0	1 (10%)	10
	VKORC1	1 (100%)	0	0	0	0	1

Fibrinogen/Factor I Deficiency

Epidemiology: Afibrinogenemia, described in 1920, affects about 1 in 1,000,000 people and represents 7% of rare bleeding disorders, with a higher incidence in women and in regions with high consanguinity. Dysfibrinogenemia and hypodysfibrinogenemia, more frequent autosomal dominant forms, have over 500 reported cases since the first description in 1958 [19].

Pathophysiology: Fibrinogen abnormalities, whether quantitative or qualitative, can lead to severe pathologies. Congenital deficiencies include afibrinogenemia, hypo-fibrinogenemia, and dysfibrinogenemia, affecting both genders. Diagnosis mainly relies on measuring antigenic fibrinogen using immunological techniques [20]. Afibrinogenemia is a complete absence of fibrinogen due to a synthesis defect, with an undetectable level (<0.1 g/L). It is the most severe form of the deficiency, leading to spontaneous and potentially serious bleeding, typically manifesting at birth or in childhood [20]. Hypofibrinogenemia is a quantitative deficiency of fibrinogen, with a reduced but detectable level (usually between 0.5 g/L and the lower limit of the laboratory). It is mostly asymptomatic, although some patients may present with post-traumatic or post-surgical bleeding [20]. Dysfibrinogenemia is a qualitative fibrinogen abnormality where the plasma level is normal, but its structure is altered, affecting its functionality. Over 50% of affected patients are asymptomatic, and diagnosis is often incidental. Some forms may be linked to hemorrhagic or thromboembolic manifestations [20].

Diagnostic approach: The biological diagnosis of fibrinogen deficiency is initially suggested by prolonged coagulation times, reflecting impaired conversion of fibrinogen to fibrin, along with a decreased functional fibrinogen activity relative to its antigenic level. In cases of afibrinogenemia, both the prothrombin time (PT) and activated partial thromboplastin time (aPTT) are typically unmeasurable (incoagulable), whereas in hypofibrinogenemia, their prolongation correlates with the concentration of circulating fibrinogen. However, to specifically assess fibrinogen functional activity, the Clauss method remains the gold standard and is widely used in clinical laboratories for diagnosing fibrinogen deficiencies. In suspected afibrinogenemia, antigenic fibrinogen testing may be performed to determine the actual protein level. It is important to emphasize that although PT and aPTT may be prolonged in such cases, they are not suitable as primary diagnostic tools due to their lack of specificity [21].

Genetic mutations: Fibrinogen deficiency is typically caused by mutations in the FGA, FGB, and FGG genes on chromosome 4, which encode the α, β, and γ chains of fibrin, respectively [22]. Most mutations causing quantitative deficiencies are primarily located in the FGA gene. These mutations can be homozygous or compound heterozygous in afibrinogenemia, and generally heterozygous in hypofibrinogenemia [21] (Tables 2, 3). The most common mutations are null mutations, including large deletions and splice site mutations. Missense mutations at the C-terminus of the B and C chains emphasize their role in fibrinogen assembly and secretion [19]. The C-terminal domain of the A chain, lacking a globular domain, can tolerate abnormalities. Most mutations lead to untranslated Ribonucleic Acid (RNA) or a truncated polypeptide that cannot assemble with the other chains [21]. Most dysfibrinogenemias are caused by missense mutations in the heterozygous state [19]. The most frequently mutated residues are arginine 35 (Arg16) in FGA exon 2 and arginine 301 (275) in FGG exon 8, where arginine is substituted by cysteine or histidine. Around 85% of dysfibrinogenemia mutations are in these two exons. Molecular screening should start with sequencing FGA exon 2 and FGG exon 8, followed by FGB exon 2, FGG exon 9, and then other exons [21]. Hypodysfibrinogenemia can result from various mechanisms. A patient may be a heterozygous carrier of a mutation affecting both fibrinogen secretion and function, or a compound heterozygote with mutations impairing fibrinogen function and biosynthesis [21].

**Table 2:** Most frequent mutations causing afibrinogenemia c-DNA: Coding DNA, T: Thymine, G:guanine, FGA: Fibrinogen alpha chain gene.

c-DNA	Gene	Type
Deletion 11 kb	FGA	Large deletion of exons 2-6
c.510+1G -> T	FGA	Splice site of intron 4

**Table 3:** Most frequent mutations causing dysfibrinogenemia c-DNA: Coding DNA, R: Arginine, C: Cysteine, H: Histidine, A: Adenine, T: Thymine, C: Cytosine, G: Guanine.

c. ADN	Gene	Exon	Nascent Protein	Mature Protein
c. 103C -> T	FGA	2	R35C	R16C
c. 104G -> A	FGA	2	R35H	R16H
c. 901C -> T	FGG	8	R301C	R275C
c. 902G -> A	FGG	8	R301H	R275H

Prothrombin/Factor II Deficiency

Epidemiology: FII deficiency is one of the rarest clotting factor deficiencies, making up only 1.5% of rare bleeding disorders (RBDs). Its global prevalence is estimated at one in 2,000,000, with a higher frequency in Latin American/Hispanic individuals. About 70% of FII deficiency cases are from regions like Barcelona, Padua, Segovia, and Puerto Rico [23]. Prothrombin deficiency follows recessive inheritance [24].

Pathophysiology: Prothrombin deficiency is divided into two types: Type I (hypoprothrombinemia), a quantitative deficiency with severely reduced prothrombin activity and antigen (<5% of normal), causing severe bleeding symptoms even without trauma. Type II (dysprothrombinemia) is a qualitative deficiency where prothrombin activity is decreased (around 10% of normal) but antigen levels remain normal or slightly reduced. Homozygous and compound heterozygous forms are symptomatic, while heterozygotes are usually asymptomatic but may bleed during surgery, childbirth, or dental extractions [25].

Diagnostic approach: In patients with hypo- or dysprothrombinemia, routine coagulation tests such as PT and aPTT are often variably prolonged. However, these global screening tests lack specificity and are insufficient to determine the exact nature of the defect. Therefore, when a prothrombin (factor II) deficiency is suspected, a specific factor II activity assay should be performed. This one-stage clotting test, considered the gold standard for diagnosing factor II deficiency, is based on the PT system and involves mixing the patient’s plasma with commercially available factor II-deficient plasma, using tissue thromboplastin as the activator. This method provides a reliable quantitative measurement of factor II activity. In cases where the factor II activity is found to be decreased, a complementary antigenic assay may be performed to evaluate the total amount of circulating prothrombin. This distinction is crucial, as hypoprothrombinemia reflects a quantitative deficiency (low activity and low antigen levels), whereas dysprothrombinemia corresponds to a qualitative defect, with normal antigen levels but reduced functional activity [26].

Genetic mutations: The human genome mutation database lists 32 mutations responsible for prothrombin deficiency. The molecular basis is heterogeneous, with mutations or polymorphisms in the F2 gene linked to both bleeding and thrombotic conditions [27]. Most identified mutations are missense (80%), with insertions/deletions (10%), nonsense mutations (6%), and splice site mutations (4%) also described [23]. Six mutations associated with prothrombin deficiency have been reported in the Indian literature, including two novel mutations: Prothrombin Vellore (p.Ala405Thr) affecting the B chain of α-thrombin, and Prothrombin Mumbai (c.G269C) causing a p.Cys90Ser substitution. Mutations in the F2 gene usually do not result in complete prothrombin absence, as this would be incompatible with life [28].

Proaccelerin/Factor V Deficiency

Epidemiology: Factor V deficiency is an extremely rare autosomal recessive disorder (one in 1,000,000 cases), with a 10-fold higher frequency in regions with high consanguinity (Middle East, Iran, Sephardic Jewish communities, India, Mediterranean basin). Around 200 cases have been reported, though this number is likely underestimated. Data is available from Iran (35 cases out of 65 million since 1998), Italy (35 cases out of 55 million since 1980), and France (54 cases in 2020). Most cases are quantitative (type I), with 25% being qualitative (type II), including FV New Brunswick, which affects factor V stability [29].

Pathophysiology: Congenital factor V deficiency is divided into two types: type I (quantitative) with undetectable functional factor V levels, and type II (qualitative) with normal or slightly decreased levels but nonfunctional. About 75% of cases are type I, and 25% are type II, with the Ala221Val (FV New Brunswick) mutation being the only studied type II variant. In both types, the absence of the prothrombinase complex prevents thrombin formation, disrupting the coagulation cascade and promoting bleeding [29].

Diagnostic approach: Laboratory evaluation plays a crucial role in the diagnosis of Factor V deficiency. In patients presenting with persistent bleeding suggestive of this disorder, initial screening includes routine coagulation tests such as bleeding time (BT), thrombin time (TT), prothrombin time (PT), activated partial thromboplastin time (aPTT), and platelet count. Since Factor V is part of the common coagulation pathway, affected individuals typically present with normal thrombin time and platelet count, while both PT and aPTT are prolonged. However, the definitive diagnosis relies on the measurement of Factor V activity levels, which is the most specific and essential test to confirm the deficiency and assess its severity [30].

Genetic Mutations: Patients with factor V deficiency may be homozygous, usually in consanguineous parents, or compound heterozygous. While severe cases are common, heterozygous patients with a dysfunctional gene copy may experience bleeding diathesis of varying severity [31]. The mutational spectrum of factor V deficiency is diverse, with over 200 cases and more than 100 genetic alterations documented, including nonsense, missense, insertion, deletion, and splice site mutations. Two-thirds of mutations are nonsense mutations, while one-third cause premature stop codons. New missense mutations primarily affect domains A and C, impairing factor V secretion and significantly reducing antigen levels [31]. Factor V deficiency is classified into type I (quantitative defects, Cross-reactive material negative) (CRM-) and type II (qualitative defects, Cross-reactive material positive) (CRM+), with type I being more common. Around 25% of cases are type II, with only one variant, Ala221Val (FV New Brunswick), studied in detail. Most other mutations, including many missense mutations, are considered type I, though many reports lack antigen measurements to confirm CRM status [31]. Most mutations causing factor V deficiency lead to CRM-like disease. Premature translation termination mutations result in mRNA degradation, and missense mutations typically cause secretion defects or intracellular degradation. For instance, FV Pro132Arg reduces secretion by 77% and specific activity by 78%. These findings suggest that the secretory pathway is highly sensitive to mutations, explaining the rarity of CRM+ mutations. Splice variants, often causing severe deficiency, typically disrupt splice sites, leading to exon exclusion or the creation of cryptic splice sites [31].

Proconvertin/Factor VII Deficiency

Epidemiology: FVII deficiency, which is inherited in an autosomal recessive manner, affects approximately 1 in 500,000 people in its severe form, although this prevalence is likely underestimated [32]. Consanguinity increases its frequency by favoring homozygosity or compound heterozygosity. The deficiency affects both males and females [32], with a frequency of severe forms estimated at 1/300,000 in Europe [33]. The clinical picture is very variable depending on the residual FVII level. Severe forms can manifest at birth with umbilical hemorrhage, in children with the loss of baby teeth, and at puberty with severe menorrhagia. Moderate forms are often diagnosed in adulthood following post-surgical or post-traumatic hemorrhagic complications [32].

Pathophysiology: Factor VII (FVII) deficiency impairs the activation of the coagulation cascade due to either reduced production or dysfunctional FVII, leading to decreased thrombin generation and fibrin formation. Clinically, this results in a variable bleeding tendency, ranging from mild to severe hemorrhagic episodes. FVII deficiency can be classified as quantitative, where both FVII activity (FVII:C) and antigen levels (FVII:Ag) are proportionally decreased, or qualitative, where FVII:Ag is normal or slightly reduced but FVII:C is disproportionately low [33]. Laboratory assessment lacks standardization, as different reagents and techniques can yield variable results, especially in the presence of certain FVII variants [33]. Notably, the severity of bleeding does not always correlate directly with FVII levels; however, severity is often categorized based on residual FVII activity: less than 5% indicates severe deficiency, 5-20% moderate, and over 20% mild [34].

Diagnostic approach: The positive diagnosis is based on biological findings, particularly a prolonged PT, suggesting a defect in the extrinsic pathway, and a normal aPTT, indicating the integrity of the intrinsic pathway. Confirmation is achieved through a specific assay of factor VII activity [35].

Genetic mutations: At the molecular level, over 250 different mutations in the F7 gene have been identified in the literature and databases, covering all mutation types: nonsense, missense, splice site mutations, deletions, insertions (with or without frameshifts), and rarely, large gene rearrangements. Missense mutations are the most frequent. Some FVII variants affect FVII:C levels depending on the type of thromboplastin used and are more common in certain regions. Additionally, six intragenic polymorphisms are known to influence FVII:C levels, adding to the complexity of F7 genotypes [33]. Several mutations cause variations in FVII:C levels based on the animal or human origin of the thromboplastin (Table 4) [36]. Typically, these mutations result in a functional defect (decreased FVII:C) but with normal antigen levels (FVII:Ag), representing qualitative deficiencies [33]. Notably, the F7: p.Arg364Gln coding for FVIIPadua and F7: p.Arg139Gln mutations, common in Africa and Southern Europe, show severely reduced FVII:C levels (<10%) when using rabbit thromboplastin but much higher levels with human-derived thromboplastins. Similarly, the rarer F7: p.Arg364Trp coding for the FVII Nagoya mutation causes severe reductions with rabbit thromboplastin FVII:C (<5%), normal levels (60%) with bovine thromboplastin, and intermediate levels (16%) with recombinant human thromboplastin. The p.Arg337His mutation, frequent in Haut-Doubs, has only been seen in compound heterozygous states and shows higher FVII:C levels with human compared to rabbit thromboplastin [33]. It is also suggested that some FVII variants could display the opposite pattern (higher FVII:C with rabbit thromboplastin), but these are rarely identified because assays are usually confirmed only from rabbit to human thromboplastin, the reverse is not true [33].

**Table 4:** Genes and chromosomes of deficient factors FGA: Fibrinogen alpha chain gene, FGB: Fibrinogen beta chain gene, FGG: Fibrinogen gamma chain gene, F2: Coagulation factor II gene, FV: Coagulation factor V gene, F7: Coagulation factor VII gene, F10: Coagulation factor X gene, F11: Coagulation factor XI gene, F13A1: Coagulation factor XIII A chain gene, F13B: Coagulation factor XIII B chain gene.

Deficit	Gene and chromosome
FI	FGA, FGB, FGG (all on 4q28)
FII	F2 (11q11-q12)
FV	F5 (1q24.2)
FVII	F7 (13q34)
FX	F10 (13q34)
FXI	F11 (4q35.2)
FXIII	F13A1 (16q24-p25), F13B (1q31-q32.1)

Stuart-Prower/Factor X Deficiency

Epidemiology: Severe congenital factor X deficiency (homozygous) is an extremely rare autosomal recessive disorder, with an incidence of one per 500,000 to 1,000,000 people [37]. According to the World Federation of Hemophilia, 2500 cases were reported globally in 2019 [38]. It is more frequent in regions with consanguinity, such as Iran (one case per 200,000; 1.3% of patients with inherited bleeding disorders), compared to 0.4% in Italy and 0.5% in the UK. Heterozygous factor X deficiency may have a prevalence of one in 500 cases [37].

Pathophysiology: In type I factor X deficiency, both functional activity and antigen levels are decreased, whereas in type II, a dysfunctional molecule is present [37]. Type I deficiency may result from secretion defects caused by point mutations in domains of the mature protein, as confirmed by expression studies [39]. Factor X deficiency is among the most severe bleeding disorders, with bleeding severity correlating with factor X levels. Most patients with factor X levels below 10% show a hemorrhagic phenotype, and those with levels below 1% experience particularly severe, early bleeding, with up to 20% developing intracerebral hemorrhages. Around 30% of heterozygous patients may present significant postoperative or spontaneous mucocutaneous bleeding. The hemostatic threshold is generally between 15% and 20% of functional factor X levels [40].

Diagnostic approach: From a biological perspective, patients typically present with a prolonged PT and an extended aPTT, reflecting a defect in the common coagulation pathway. Liver function tests are strictly normal. In some cases, reduced levels of factor X are observed; this is confirmed by performing a specific factor X activity assay, which is essential to distinguish between isolated Factor V deficiency and combined deficiencies [41].

Genetic mutations: The Gly204Arg substitution causes severe type I factor X (FX) deficiency by inducing structural changes and a secretion defect due to retention in the trans-Golgi and late endosome. It destabilizes the disulfide bond between the FX chains, reducing interaction between the Cys201-Cys206 loop and the region connecting the Second epidermal growth factor-like domain (EGF2) and serine protease domains [39]. DNA analysis revealed a compound heterozygous state: the known mutation of Gly244 to Arg (p.G244R) in exon 6 (paternal origin) and a novel mutation of Gly435 to Ser (p.G435S) in exon 8 (maternal origin). The p.G244R mutation, previously reported as FX Debrecen, produces a nonsecretable protein. Glycine at amino acid position 435 in the C-terminal region is completely conserved in the trypsin-like serine protease family. In a three-dimensional structural model of FX, Gly435 was located in the 11th β-chain and buried at the rear of the catalytic pocket, and its substitution with serine is predicted to disrupt the structure and prevent secretion. Both mutations likely cause type I FX deficiency, characterized by undetectable plasma activity and antigen levels, associated with a severe bleeding phenotype [42].

Rosenthal/Factor XI Deficiency

Epidemiology: Factor XI deficiency is a rare hereditary disorder (≈1 in 1,000,000 worldwide), but more frequent among Ashkenazi Jews (8% heterozygous) and certain groups like Iraqi Jews, French Basques, and a community in northeast England. Endogamy contributes to the increase in severe forms. In the UK, it accounts for approximately 3% of bleeding disorders, mostly in individuals without Jewish ancestry [43]. Though typically autosomal recessive, dominant inheritance can occur [43].

Pathophysiology: Factor XI deficiency primarily results from FXI gene mutations, causing quantitative or, more rarely, qualitative defects. It is classified into two types: quantitative deficiency (CRM-), with concordant reductions in FXI:Ag and FXI:C, and qualitative deficiency (CRM+), with normal or slightly reduced FXI:Ag but significantly decreased FXI:C due to dysfunctional circulating FXI. Most mutations are associated with the CRM- phenotype. FXI is crucial for thrombin generation and fibrinolysis regulation via thrombin-activated fibrinolysis inhibitor (TAFI) activation; its deficiency leads to impaired clot formation and stability, promoting bleeding [44]. The deficiency arises from reduced or absent polypeptide synthesis, inability to form dimers, and non-excretion of normal homodimers [45]. FXI levels below 30% usually indicate biallelic mutations, while levels between 30-55% suggest monoallelic involvement, though these thresholds are subject to revision [40].

Diagnostic approach: Diagnosis is established based on unexplained bleeding episodes, a positive family history, or preoperative laboratory evaluation revealing an isolated prolonged aPTT that corrects upon mixing studies, with normal PT, TT, and fibrinogen levels. In addition to mixing studies, the measurement of Factor XI activity is essential for confirming the diagnosis and is routinely performed as part of the initial screening [46].

Genetic mutations: Factor XI deficiency results from various mutations in the FXI gene, including missense, nonsense, splice site alterations, and insertions/deletions [45]. Quantitative deficiency (CRM-) is classified into three categories based on the effect of the mutations. The first group includes mutations like Glu117X and Ala91Thr, which disrupt protein synthesis and lead to selective degradation of the mutant mRNA via nonsense-mediated decay (NMD). The second group involves mutations in the A4 domain, such as Phe283Leu, which prevent dimer formation and cause intracellular retention of FXI monomers. The third group includes mutations like Gly400Val, Trp596Ser, Ser225Phe, and Cys398Tyr that form non-secretory homodimers, impairing secretion of heterodimers formed with the normal FXI allele, resulting in a dominant phenotype and intracellular retention of the mutated proteins. Other mutations, including Cys58Tyr, Tyr427Cys, Cys527Tyr, Val20Ala, and Glu297Lys, affect protein secretion due to a folding defect. The molecular basis of qualitative FXI deficiency (CRM+) remains less well defined, though it involves dysfunctional FXI [44]. One study found heterozygotes usually do not show a bleeding tendency, supporting autosomal recessive inheritance, while some heterozygotes with low FXI levels exhibit a dominant-negative effect: the mutated protein formed a heterodimer with that from the normal allele, thus impairing FXI secretion [44]. Homozygotes and compound heterozygotes have a severe deficiency, while single heterozygotes show partial deficiency [44].

Fibrin-Stabilizing/Factor XIII Deficiency

Epidemiology: Congenital factor XIII deficiency is an extremely rare bleeding disorder inherited in an autosomal recessive manner, with over 500 cases reported worldwide since 1960, and a frequency of about one in four million births [47]. It accounts for 6% of rare bleeding disorders in France [48], and is more prevalent in consanguineous populations, such as in Iran [49].

Pathophysiology: Factor XIII deficiency prevents fibrin monomer polymerization, leading to an unstable clot that is easily degraded, causing a bleeding syndrome [50]. Standard coagulation tests (PT, aPTT, fibrinogen, platelet count, bleeding time) are normal, complicating diagnosis [51], which is confirmed by specific dosage in the presence of clinical signs [48]. Menorrhagia affects 30-60% of women, and pregnant women have a 66% miscarriage rate [52]. Factor XIII also plays a key role in wound healing and fertilized egg implantation [50]. FXIII deficiency is classified into three subtypes: major (undetectable activity), moderate (activity <30%, minor bleeding), and mild (activity 30-70%, often asymptomatic) [47].

Diagnostic approach: Functional assays of factor XIII (FXIII) remain the gold standard for diagnosis, but their lack of standardization and specificity limits their use. Among these, the clot solubility test is non-standardized and insensitive; the ammonia release test is rapid but has low sensitivity; the amine incorporation test is sensitive but time-consuming and not automatable; and the chromogenic method, although well-automated and widely used in laboratories, lacks sensitivity for detecting very low FXIII levels. Antigenic assays, necessary for typing the deficiency, use immunological techniques measuring the FXIII-A and FXIII-B subunits or the FXIII-A2B2 complex. The Enzyme-Linked Immunosorbent Assay (ELISA) targeting the FXIII-A subunit is the most common and correlates well with functional activity, while an automated latex particle-based method shows good correlation and sensitive detection (<4%). The rarity of congenital deficiency raises questions about the choice of the appropriate technique, especially since screening is crucial in clinical situations with a high risk of severe bleeding [48].

Genetic mutations: The F13A1 gene, encoding FXIII-A, is located on chromosome 6p24-25 and contains 15 exons and 14 introns [47]. Nine polymorphisms have been identified, seven causing amino acid substitutions, with variable frequencies across populations. The Val34Leu polymorphism, present in about 20% of Europeans, alters fibrin clot structure depending on fibrinogen levels and is associated with reduced risks of venous thromboembolism and myocardial infarction, but increased risks of ischemic stroke and intracerebral hemorrhages [47,53]. The Phe204 allelic variant of the Tyr204Phe polymorphism correlates with reduced FXIII levels and increased miscarriage risk [47]. The Leu564 allele of the Pro564Leu polymorphism decreases plasma FXIII levels but increases in its activity, though findings remain to be confirmed [47]. The F13B gene, encoding FXIII-B, is located on chromosome 1q31-32 and consists of 12 exons and 11 introns. Its terminal region promotes isoform formation, leading to three main phenotypes: FXIII-B*1 (Europe), FXIII-B*2 (Africa), and FXIII-B*3 (Asia) [47]. FXIII-B*2 is linked to higher FXIII activity, while FXIII-B*3 is associated with lower levels. The His95Arg polymorphism, typical of FXIII-B*2, is frequent in Africa (≈60%) but absent in Asia, and it enhances dissociation of the FXIII-A2B2 complex, possibly affecting thrombosis risk, and would interact with the Leu34 variant of the A subunit, reducing myocardial infarction risk in women under estrogen therapy. The c.1952+144 C>G polymorphism, corresponding to FXIII-B*3, is common in Asia (>60%) and leads to a 15-amino-acid elongated protein, with unclear functional consequences [47].

Combined Factor V and Factor VIII Deficiency

Epidemiology: Combined factor V and VIII deficiency is a rare autosomal recessive disorder, with a prevalence of 1 in 1,000,000, rising to 1 in 100,000 among Sephardic Jews and non-Jewish Iranians. It is frequent in the Mediterranean (Tunisia, Italy, Algeria, Turkey), Asia, Eastern Europe, and the Americas. No differences in factor levels are observed by sex or blood type. In Morocco, only two cases have been reported [54].

Pathophysiology: Mutations in the MCFD2 gene can cause a greater reduction in factor V and VIII levels, with more severe bleeding symptoms, compared to mutations in LMAN1. These deficiencies result from genetic defects affecting proteins critical for the intracellular transport of factors V and VIII. Mutations in LMAN1/MCFD2 disrupt the formation of their complex, which is essential for exporting these factors out of the cell, making them unavailable for coagulation. While some families show no mutations in either gene, suggesting a possible third locus, the LMAN1-MCFD2 complex remains the main mechanism identified in the majority of cases of Combined factor V and factor VIII deficiency (DF5F8). This complex is the first and only known cargo receptor for factors V and VIII. Bleeding symptoms are usually mild and similar to isolated deficiencies of factor V or VIII [54].

Diagnostic approach: Patients present with bleeding symptoms of variable severity, typically with prolonged PT and aPTT, showing a ratio between 1.2 and 2.5, which is corrected by mixing with normal plasma. Specific assays of coagulation activity reveal a combined deficiency of factors V and VIII, with residual activity levels ranging between 5% and 30%. Antigenic assays are not strictly required for the diagnosis [54].

Genetic mutations: Combined factor V and VIII deficiency is linked to mutations on chromosome 18q21, between markers D18S849 and D18S64. Over 100 mutations in LMAN1 and 45 in MCFD2 have been identified, mainly involving insertions/deletions, nonsense mutations, and splice site defects, all causing loss of protein function. Some mutations affect regulatory regions, such as promoter deletions. A founder effect has been observed for certain mutations, like M1T (c.2T>C) in Italian families and c.89-90insG and c.1149+2T>G in Jewish patients. This founder effect is particularly observed in populations where the prevalence of the combined deficiency is higher, with an increased rate of consanguinity. The mutations responsible for this deficiency can affect different regions of the affected genes and are classified into five categories: nonsense mutations, leading to premature termination of translation; deletions and insertions, resulting in a frameshift and a non-functional truncated protein; mutations affecting the first methionine codon, as observed for LMAN1, preventing translation initiation; splice site abnormalities, disrupting mRNA splicing and thus altering protein structure; and missense mutations, which modify the amino acid sequence without necessarily completely abolishing protein function. These mutations, by altering the function of LMAN1 and MCFD2 proteins, compromise the intracellular transport of factors V and VIII, leading to their plasma deficiency and, consequently, hemorrhagic manifestations of varying severity [55].

Vitamin K-Dependent Combined Clotting Factor Deficiency

Epidemiology: Vitamin K-dependent combined clotting factor deficiency is an extremely rare hereditary disorder, with fewer than 30 affected families identified worldwide. It is inherited in an autosomal recessive manner, requiring both parents to carry a mutation for the disease to manifest in their child. Due to their rarity, estimating the geographic distribution and prevalence of vitamin K-dependent combined clotting factor deficiencies is challenging. They are more common in populations with frequent consanguineous marriages. In France, the prevalence is estimated at 1 case per 1,000,000 individuals, though this may be an underestimate due to clinical and genetic heterogeneity [40].

Pathophysiology: Hereditary vitamin K-dependent coagulation factor deficiency (VKCFD) is a rare disorder characterized by a simultaneous deficiency of coagulation factors II, VII, IX, and X, as well as anticoagulant proteins: protein C (PC), protein S (PS), and protein Z (PZ) [56]. Two phenotypes of vitamin K-dependent coagulation factor deficiency are described: VKCFD1, associated with mutations in the GGCX gene, and VKCFD2, linked to mutations in the VKORC1 gene. These mutations impair the vitamin K cycle, reducing γ-carboxylation of Gla proteins and hemostatic function. VKCFD1 is often associated with a pseudoxanthoma elasticum-like disorder (PXE-like disorder), which involves developmental, skeletal, and dermatological abnormalities. Thirteen mutations are specifically linked to this PXE-like syndrome [40].

Diagnostic approach: VKCFD is primarily diagnosed through laboratory testing. It should be suspected in the presence of reduced PT, with or without prolonged aPTT. Coagulation factor assays typically reveal decreased levels of vitamin K-dependent factors evaluated by PT (factors II, VII, and X), as well as factor IX. To support the diagnosis, levels of natural anticoagulants protein C and protein S are also found to be decreased. Although protein Z may also be reduced, its measurement is not approved for diagnostic use and is currently limited to research laboratories only. Factor levels vary significantly among patients: factor II can range from 2% to normal, factor VII from <1% to normal, factor IX from 4% to normal, and factor X from 2% to 33% [57].

Genetic mutations: VKCFD arises from mutations in two genes: GGCX (VKCFD type 1) and VKORC (VKCFD type 2). These genes are crucial for protein carboxylation, which activates coagulation factors and inhibitory proteins. Impaired carboxylation reduces the activity of Gla proteins, leading to hemostatic dysfunction. GGCX, identified in 1991, has several known mutations, including a 14-base pair deletion in intron 1, disrupting protein expression and potentially altering enzyme function. VKCFD type 1 is often caused by compound heterozygosity, with two missense mutations in the GGCX gene, affecting protein carboxylation. VKCFD type 2 is linked to mutations in the VKORC gene, encoding subunit 1 of the vitamin K-epoxide reductase (VKOR) complex, essential for vitamin K reduction and regeneration for carboxylation. VKOR was identified 34 years ago, but was only isolated and characterized in 2004 using reverse genetics and expression cloning. A mutation, 292C > T in the VKORC1 gene, responsible for VKCFD, was identified in Lebanese, German, and Italian families. This mutation causes an amino acid change (Arg98Trp) near the vitamin K binding site. It affects both VKCFD development and the response to vitamin K, notably influencing warfarin resistance, which has important implications for the clinical management of affected patients [58].

Rare coagulation factor deficiencies exhibit diverse profiles depending on the affected factor. Most follow an autosomal recessive inheritance pattern and are associated with various genetic mutations, including missense, nonsense, insertion, deletion, and splice site variants. They are characterized by variable clinical manifestations, and diagnosis relies on specific functional and antigenic assays tailored to each deficient factor (Table 5) [59].

**Table 5:** Comparative summary table of rare coagulation factor deficiencies AR: Autosomal recessive, AD: Autosomal dominant, PT: Prothrombin time, aPTT: Activated partial thromboplastin time, FGA: Fibrinogen alpha chain gene, FGB: Fibrinogen beta chain gene, FGG: Fibrinogen gamma chain gene, F2: Coagulation factor II gene, F5: Coagulation factor V gene, F7: Coagulation factor VII gene, F10: Coagulation factor X gene, F11: Coagulation factor XI gene, F13A1: Coagulation factor XIII A chain gene, F13B: Coagulation factor XIII B chain gene, LMAN1: Lectin, Mannose-Binding 1, MCFD2: Multiple Coagulation Factor Deficiency Protein 2, GGCX: Gamma-glutamyl carboxylase, VKORC1: Vitamin K epoxide reductase complex subunit 1.

Factors	Inheritance	Common Mutations/ Genetic Cause	Clinical Manifestations	Diagnostic Assays
Factor I	AR/AD	Mutations in FGA/ FGB / FGG genes (afibrino‑/hypo‑/dys‑)	Intracranial hemorrhage, Umbilical cord bleeding, Hemarthrosis, Menorrhagia, Epistaxis	Clauss assay, PT and aPTT prolonged
Factor II	AR	Hypoprothrombinemia or dysprothrombinemia (F2 gene)	Menorrhagia, Hematoma, Postpartum hemorrhage, Epistaxis	Prolonged PT/aPTT Prothrombin activity/antigenic assays
Factor V	AR	F5 gene missense, nonsense, insertion, deletion and splice site mutations	Epistaxis, Menorrhagia, Gastrointestinal bleeding, Hem-arthrosis, Postoperative bleeding	Prolonged PT/aPTT Factor V activity/antigenic assays
Factor VII	AR	F7 gene missense, nonsense, insertion, deletion and splice site mutations	Hematoma, Hemarthrosis, Postoperative bleeding, Intracranial hemorrhage, Menorrhagia	Prolonged PT/ normal aPTT; Factor VII activity/antigenic assays
Factor X	AR	F10 gene mutations	Intracranial hemorrhage, Umbilical cord bleeding, Easy bruising Menorrhagia, Epistaxis, Gastrointestinal bleeding	Prolonged PT/aPTT Factor X activity/antigenic assays
Factor XI	AR/AD	F11 gene missense, nonsense, insertion, deletion and splice site mutations	Postsurgical bleeding, Epistaxis Postpartum hemorrhage, Gum bleeding, Easy bruising, Menorrhagia	Prolonged aPPT/ normal PT; Factor XI activity/antigenic assays
Factor XIII	AR	F13A1 / F13B gene mutations	Intracranial hemorrhage, Umbilical cord bleeding, Recurrent miscarriage	Normal PT/aPTT; FXIII activity assay; urea clot solubility test
Factor V+Factor VIII	AR	LMAN1 or MCFD2 mutations	Easy bruising, Epistaxis, Postoperative bleeding, Gingival bleeding	Combined factor V and VIII assays
Vitamin K-dependent Factors	AR	Mutations in VKORC1 / GGCX	Intracranial hemorrhage, Umbilical cord bleeding, Ecchymosis, Mucocutaneous bleeding, Posttraumatic hemorrhage	PT prolonged with or without prolonged aPTT, low levels of all four vitamin K-dependent factors

Clinical overview

**Figure 1:**
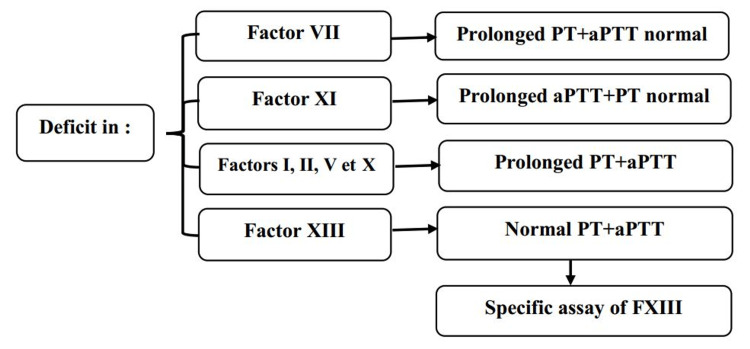
Schematic of the biological diagnosis of rare coagulation factor deficiencies PT: Prothrombin time, aPTT: Activated partial thromboplastin Time.

Rare coagulation factor deficiencies are most often discovered following spontaneous or excessive bleeding triggered by minor trauma, surgery, or dental procedures. They may also be revealed through family history, abnormal menstrual bleeding, genetic screening in at-risk families, or during neonatal or prenatal evaluations. Prolonged or difficult-to-control bleeding is a major warning sign that prompts further investigation. Bleeding manifestations vary considerably depending on the deficient factor and also between individuals with the same genetic anomaly. Heterozygous individuals are generally asymptomatic, which makes clinical assessment sometimes subjective and complex. To better address this heterogeneity, a European expert group proposed a four-level classification of bleeding severity, ranging from asymptomatic to major hemorrhage. The most frequent symptoms include mucosal bleeding, menorrhagia, and postoperative hemorrhage. In contrast, hemarthroses and deep hematomas are primarily associated with factor II and X deficiencies. Bleeding from the umbilical stump shortly after birth is highly suggestive of factor XIII deficiency or afibrinogenemia. According to the European Network of Rare Bleeding Disorders, there is a strong correlation between bleeding severity and factor levels in fibrinogen, factor X, and factor XIII deficiencies. A weaker correlation is observed for factor V and VII deficiencies, and no correlation is noted for factor XI deficiency [3]. The biological diagnosis of rare coagulation factor deficiencies begins with a standard hemostasis panel, including prothrombin time (PT), activated partial thromboplastin time (aPTT), and fibrinogen level. These initial tests assess the major coagulation pathways (extrinsic, intrinsic, and common) and help identify potential abnormalities (Figure 1). When abnormal results are observed, specific coagulation factor assays are required to determine precisely which factor is deficient. The combined interpretation of PT, aPTT, and fibrinogen values thus represents the essential first step in guiding the diagnostic pathway [60].

Identification of rare coagulation factor deficiencies at the hematology laboratory of CHU Ibn Rochd, Casablanca, Morocco

At the hematology laboratory of CHU Ibn Rochd, the identification of rare coagulation factor deficiencies is based on blood tests performed in the context of bleeding symptoms or as part of a preoperative check-up. These tests include a standard hemostasis workup, particularly PT, aPTT, and specific plasma factor assays. However, molecular analysis is not performed locally in Morocco. In cases with a severe clinical presentation requiring genetic counseling, genomic DNA is extracted on-site from peripheral blood collected in Ethylenediaminetetraacetic acid (EDTA) tubes using the Qiagen kit. The extracted DNA is then sent to specialized laboratories abroad for NGS.

## Conclusions

Rare coagulation factor deficiencies represent a significant diagnostic and therapeutic challenge due to their clinical heterogeneity and the limited availability of specialized diagnostic tools, particularly in low-resource countries. Molecular characterization plays a crucial role not only in elucidating the underlying pathophysiological mechanisms, such as nonsense, missense, frameshift, or splice-site mutations in the genes encoding coagulation factors, but also in refining patient classification and assessing bleeding risk. The identification of specific mutations enhances our understanding of genotype-phenotype correlations and underscores the importance of genetic counseling, especially in populations with a high rate of consanguinity. These molecular findings pave the way for more targeted therapeutic strategies, including individualized replacement therapies, prophylactic protocols, and, in the longer term, gene therapies. Optimal patient care relies on a multidisciplinary approach involving hematologists, molecular biologists, and genetic counselors to ensure accurate diagnosis, appropriate treatment, and long-term follow-up. Finally, continued research and international collaboration are essential to overcome current diagnostic barriers and to improve access to molecular tools and innovative therapies for these rare bleeding disorders.
